# Synthesis and Characterization of a Hybrid Cement Based on Fly Ash, Metakaolin and Portland Cement Clinker

**DOI:** 10.3390/ma13051084

**Published:** 2020-02-29

**Authors:** Adriagni C. Barboza-Chavez, Lauren Y. Gómez-Zamorano, Jorge L. Acevedo-Dávila

**Affiliations:** 1 Facultad de Ingeniería Mecánica y Eléctrica (FIME), Universidad Autónoma de Nuevo León (UANL), San Nicolás de los Garza 66455, Mexico; adriagnicbc@gmail.com; 2 Corporación Mexicana de Investigación de Materiales, Saltillo 25290, Mexico; jacevedo@comimsa.com

**Keywords:** alkali-activated binders, hybrid cement, fly ash, metakaolin, mechanical properties

## Abstract

Hybrid cement has become one of the most viable options in the reduction of CO_2_ emissions to the environment that are generated by the cement industry. This could be explained by the reduction of the content of clinker in the final mixture and substitution of the remaining percentage with supplementary cementitious materials with the help of an alkaline activation. Following that, properties that are provided by an Ordinary Portland Cement and of a geopolymer are mixed in this type of hybrid material and could be achieved at room temperature. Thereafter, the main objective of this research was to synthesize hybrid cements reducing the clinker content of Portland Cement up to 20% and use metakaolin and fly ash as supplementary cementitious materials in different proportions. The mixtures were alkaline activated with a mixture of sodium silicate and sodium hydroxide, calculating the amounts according to the percentage of Na_2_O that is present in each of the activators. The samples were then characterized using Compressive strength, X-ray diffraction, Fourier Transform Infrared Spectroscopy, and Scanning Electron Microscopy with energy-dispersive X-ray spectroscopy. The results indicated that the hybrid cements have similar mechanical properties than an Ordinary Portland Cement, and they resulted in a dense matrix of hydration products similar to those that are generated by cements and geopolymers.

## 1. Introduction

The cement industry is currently facing challenges that are related not only to the improvement of its manufacturing processes, but also to achieving the reduction of the environmental impact generated by the manufacture of Ordinary Portland Cement (OPC). In this process, large emissions of several greenhouse gases into the environment are involved, where CO_2_ is the most important, and is the main responsible for global warming. Nowadays, OPC is the main material that is used in the construction industry [1], and its production has a considerable impact on the emission of greenhouse gases into the environment, representing between 5–7% of CO_2_ emissions worldwide. In addition, cement plants consume high amounts of primary energy, which is estimated in 3% of the worldwide consumption [2]. These CO_2_ emissions are caused by the combustion that is necessary to reach temperatures of around 1450 °C (30–35%) and the decarbonation or decomposition of limestone to produce clinker (50–60%) and the materials transport (around 10%) [3]. It should be noted that eliminating CO_2_ emissions into the environment from the decomposition of limestone is mainly impossible, since this process is necessary for the manufacture of OPC clinker (CK). However, these emissions can be reduced while using waste materials as raw material for the clinker or by using final substitute materials for OPC. These materials that serve as partial or total substitutes are called supplementary cementitious materials (SCMs) [4], of which the best known are ground granulated blast furnace slag (GGBFS), fly ash (FA), silica fume (SF), and calcined clays. The use of these materials can be a viable solution for the reduction of CO_2_ emissions [5]. Current cement consumption is projected to be double by 2050, and the use of these materials could highly reduce CO_2_ emissions to the environment [4]. Following that, a model of binders is currently being studied, which consists of adding to the mix of supplementary materials a very low portion of CaO in the form of clinker, to obtain a setting at room temperature [1]. During the past decades, alkali-activated and blended cement have attracted strong interest worldwide, due to their advantages of low energy cost, high strength, good durability, and sustainability. A major incentive for the further development of such cements is generated by the great quantity of annual generation of wastes, which cause a need to find new uses for them. The main raw materials used for this proposal are FA, GGBFS, and metakaolin (MK), in which activators, like water glass and sodium hydroxide, are used. Among the alkaline cements, two large groups are known: (a) calcium and silicon-rich activated materials (Me_2_O–MeO–Al_2_O_3_–SiO_2_–H_2_O system); here, the activation of GGBFS (SiO_2_ + CaO > 70%) corresponds, under relatively mild alkalinity conditions [6]. A hydrated calcium aluminosilicate gel (C–A–S–H) is the main hydration product generated, similar to the gel generated in the hydration of a OPC (C–S–H gel), but with a lower CaO/SiO_2_ ratio, generally between 0.9–1.2, compared to the CaO/SiO_2_ ratios of the C–S–H gel which are between 1.5–2.2 [2]; and (b) these are materials activated mainly by aluminum and silicon (Me_2_O–Al_2_O_3_–SiO_2_–H_2_O system) and low CaO contents, such as meta-kaolin or F-type FA (from coal-fired power plants). This group requires high alkalinity solutions and curing temperatures between 40 and 150 °C [7,8,9]. The main hydration product is an amorphous alkaline silicoaluminate gel, called N–A–S–H gel, which contains silicon and aluminum tetrahedrons that are distributed at random along polymer chains that intertwine, forming a three-dimensional structure [10,11].

Nevertheless, it is possible to add a third group, where the properties of the two groups that are mentioned above are mixed. Recent research shows that, when a quantity of calcium, which might be in the form of additional OPC clinker, is added to the starting mixture, the material hardens at room temperature without the need to raise the temperature in the initial setting [1]; this is known as *hybrid cements*. Hybrid cements fall into the category of low carbon footprint cements, combining the positive properties of OPC with the properties of alkali-activated materials or geopolymers [12], generally resulting in a material with good mechanical properties and high durability [13]. As a result, hybrid cements are generated while using an alkaline activation of low clinker cements (20–30%) with an activator added in proportions of approximately 5–10% (depending on the type of raw material); the rest of the mixture (65–75%) is composed of SCMs [14].

Alkaline activation and geopolymerization are technologies that allow for the use of wastes with reactive species, such as SiO_2_ and Al_2_O_3_, which are inadequate in other industries. The application of these technologies helps to promote the use of waste or industrial by-products, thus benefiting the environment and helping the sustainability [15]. By adding the alkaline activator to the hybrid cement, its reactivity potentially increases, allowing for the use of large quantities of materials that are considered to be of low quality [16].

Fly ash is a by-product of the burning of carbon in thermoelectric plants [16], it has the ability to react with calcium hydroxide, product of the hydration process of OPC, and generate a pozzolanic reaction to produce more C–S–H, providing greater strength to the material, this makes it a material widely used along with OPC [17]. The by-products of the coal industry, such as FA, which are pozzolanic aluminosilicate materials with an amorphous fraction of approximately 70%, are among the materials considered to be of low quality [18]. In contrast to FA, MK is not a by-product of the industry, and a specific process must be carried out to obtain it [19]; this process consists of calcining the kaolin at a specific temperature to create a disorder in the layers of silica and alumina and, thus, generate a material with high pozzolanic reactivity [20], it should be noted that the kaolin calcination temperature is the most important parameter, since the reactivity of the resulting material is completely dependent on this parameter [20,21]. According to previous studies, the presence of MK as a replacement for cement improves the mechanical properties of mortars and concretes as a result of their pozzolanic activity [22].

The hydration mechanism of hybrid cements is not yet fully defined; however, Yip et al. [23] indicated the coexistence of geopolymer gel and hydrated calcium silicate at early ages when a mixture of GGBFS and MK is alkali-activated. The coexistence of both gels depends on the alkalinity of the solution and the MK/GGBFS ratio. Thus, he observed that the formation of the C–S–H gel together with the polymer gel only occurs in low alkalinity systems; while, in the presence of NaOH, the polymer gel was the predominant phase, with small calcium precipitates that were dispersed. Palomo et al. [24] reported that, in hybrid cements using OPC and FA, the reaction products were a mixture of amorphous C–S–H and N–A–S–H gels. These authors concluded that, in the case of hybrid cements, different hydration patterns are followed, depending on the concentration of OH– and the silica that can be dissolved in the medium used [25]. Additionally, Garcia-Lodeiro et. al. [26] analyzed synthetic gels to explore the effects of a constituent of one gel on another, obtaining, as a result, that the composition and structure of the C–S–H gel is affected in high pH media and in the presence of aqueous aluminates. In this way, calcium modifies the N–A–S–H gel, partially replacing sodium and generating a new amorphous gel, known as C–(N)–A–S–H.

Based on the above, in this research work it is proposed to synthesize hybrid cements by reducing the percentage of OPC clinker (less than 30%) and use MK and FA as SCMs, which were activated with an alkaline solution that was composed of sodium silicate and sodium hydroxide, and in this way obtain mechanical properties that are similar to those that were reported for OPC. Following from that, the main contribution of this investigation was the synthesis and characterization of those hybrid cements at room temperature, which was not reported yet, as described below.

## 2. Materials and Methods

The precursors used in this investigation were: (a) fly ash from a thermoelectric plant in Nava, Coahuila, Mexico, which was used in a particle size under 75 μm, (b) metakaolin, which was calcined in a kiln at 800 °C for 8 h to achieve the de-hydroxylation of the material and, thus, achieve the disorder between the layers of silica and alumina necessary to increase the reactivity of the material, and (c) clinker from an OPC (Cemex, Monterrey, Mexico), which was ground to a particle size under 75 μm. For the preparation of the alkaline solution, bi-distilled water, sodium hydroxide, and commercial water glass were used (sodium silicate, from Silicatos solubles, Monterrey, Mexico). Table 1 and Table 2 show the chemical composition of each of the precursors and activators, respectively.

The alkaline solution was prepared while calculating the percentage of Na_2_O present in each of the activators as, sodium hydroxide (NaOH) and water glass. After several preliminary tests, it was decided to use 16% and 18% of Na_2_O equivalent. Table 3 shows the load of the precursors, where the clinker content was a set parameter as well as the w/s ratio for all systems: 20% and 0.65, respectively. The alkaline solution was added to the homogenized powders (precursors) at room temperature, and then the pastes were poured into 50 mm cubic molds. After a setting time of 24 h, the samples were demolded and immersed in distilled calcium hydroxide saturated water, into sealed at room temperature, until curing periods of 3, 7, 14, 28, and 90 days.

After the hydration periods, the samples were characterized by means of compressive strength; four specimens were extracted from the containers and tested where the average was reported. Afterwards, carefully taken pieces of the solid fractions of the samples were crushed and submerged in isopropyl alcohol (CTR scientific, Monterrey, Mexico) for 24 h and then dried in an oven (Sheldon Manufacturing, INC, Monterrey, Mexico) at 50 °C for another day in order to stop the hydration reactions, for later characterization. After drying, the samples were ground to obtain a powder with particle size under 75 μm. This powder was used to analyze the samples while using the following techniques of characterization: X-ray diffraction (XRD, Panalytical, Monterrey, Mexico) and Fourier-transform infrared spectroscopy (FT-IR, Perkin Elmer, Monterrey, Mexico), for the analysis by scanning electron microscopy (SEM, JEOL, Monterrey, Mexico) solid cold mounted pieces were analyzed. As a complementary technique, qualitative X-ray diffraction (XRD) analyses were carried out on the powdered samples while using a Bruker-D8 Advance diffractometer with Cu-Kα radiation (λ = 1.54). The samples were scanned on a rotating stage between 5-to-70° 2θ; with a step scan of 0.021 and a dwell time of 1 s. The time that was required for the acquisition of X-ray diffraction pattern was around 35 min. X-ray structure data for known compounds were sourced from the literature or standard databases (ICSD, JCPDS). Additionally, the characteristic bands of alkali-activated binders were analyzed by Fourier transform infrared spectroscopy (FT-IR), preparing pellets of such powders by mixing and pressing the samples with KBr to obtain the spectra (Perkin Elmer model Paragon 1000PC). Backscattered electron images in scanning electron microscopy (SEM) and Energy-dispersive X-ray spectroscopy (EDS, EDAX Monterrey, Mexico) were used to analyze the microstructure. Specimens were cold mounted in epoxy resin, ground, polished, and gold-coated prior to the analysis in SEM in order to analyze the microstructure.

## 3. Results

### 3.1. Compressive Strength

The compressive strength results showed in this section are of all the systems that are described in Table 3, cured at room temperature and tested at different periods of 3, 7, 14, and 90 days, with two percentages of alkaline activators. As mentioned above, the results were estimated as the average of four samples for each mixing system, the standard deviation showed a maximum of 2 MPa.

The best mechanical strength results were obtained at early ages in the systems with higher MK content; this assures that the amount of activator used is enough to activate MK at early age. It can be observed that the systems have a much slower increase in strength in initial stages (3–14 days), which is a characteristic of the replaced cements according to Barnett et. al. [27]. It has been reported that pozzolanic particles act as nucleation sites promoting the formation of C–S–H gel, as well as the densification of the cementitious matrix and reduction of porosity, besides improving fluidity through the effect of FA, which limits porosity [28,29]. The systems activated with 18% of Na_2_O that present greater strengths are the systems with a greater content of FA, this is confirmed by past investigations, where they indicated that a high alkaline media is required to obtain higher mechanical strengths [12]. All systems of 16% and 18% activator, synthesized and tested at 28 days, exceeds 20 MPa (the minimum compressive strength requirement of an OPC 20R), however, mixtures with 20FA-60MK reached strengths greater than 30 MPa when 16% Na_2_O was used and, when 18% Na_2_O was used, the system with compressive strength higher than 30 MPa was the 30FA-50MK system.

(a) Systems activated with 16% Na_2_O

**Figure 1:**
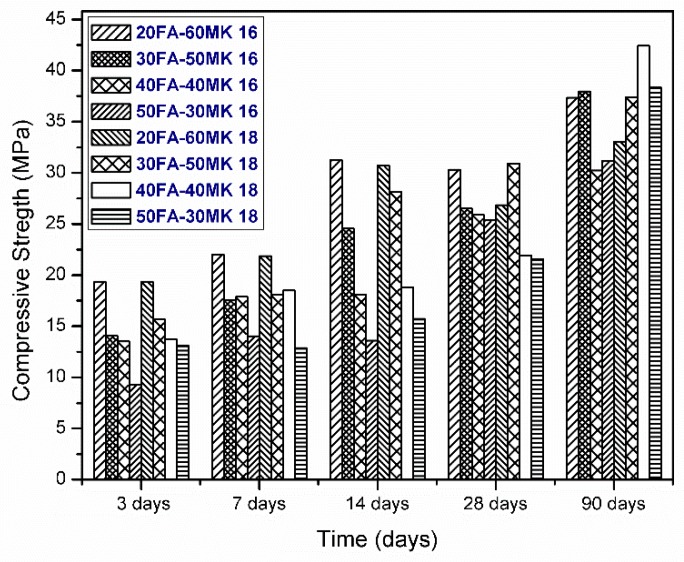
Compressive strength of systems activated with 16% and 18% Na_2_O.

Two reference systems were taken and used as controls to compare the behavior of the rest of the systems, which have 20% clinker and the rest is FA or MK, the amount of activator was the same for all of the samples. In Figure 1, it can be seen how the mechanical strength results decrease when using 16% Na_2_O and increasing the amount of FA. Nevertheless, when compared to the system, where only FA was used, the compressive strength results were considerably higher. When compared to the system with the only MK, the strengths at early age are better than the ternary systems. However, at 90 days, the strengths of the ternary systems exceed those of the binary systems, therefore, it can be concluded that the combination of FA and MK provides better strengths at later ages when this percentage of the activator is used. It can be observed that the systems have a much slower increase in strength in the initial stages (3–14 days), which is a characteristic of the blended cements with SCMs, according to Barnett et. al. [27]. It has been reported that pozzolanic particles of small sizes could act as nucleation sites promoting the formation of more C–S–H gel, as well as densifying the cementitious matrix and, thus, reducing porosity (as a micro filler effect), in addition, to improve the fluidity through the addition of FA, which also could reduce porosity [28,29].

(b) Systems activated with 18% Na_2_O

Otherwise, when 18% of Na_2_O was used, the systems with greater amounts of FA showed greater strength at 90 days when compared to the ternary systems with less amount of FA, thus the presence of more of Na_2_O favored the hydration reactions of FA, as reported before for high amount of alkaline activator [12]. When compared with the 80 MK binary system, it can be seen that the compressive strength results of this system were higher at 90 days than those of the ternary systems, approaching those of the 40FA-40MK system.

Fernandez et al. [11] studied ternary mixtures while using OPC clinker, MK and GGBFS, where alkaline activators were Na_2_CO_3_ and K_2_CO_3_, obtaining values of compressive strength at 28 days of curing with Na_2_CO_3_ of 23.17 MPa and with K_2_CO_3_ of 20.96 MPa. Garcia Lodeiro et al. [1] showed results of hybrid cements that were similar to those presented in this investigation, in which they activated 80% FA and 20% CK obtaining compressive strength values of around 30 MPa, in the same way, a ternary system with 40% MK, 40% GGBFS, and 20% CK was analyzed, with a compressive strength at 28 days of curing of 23 MPa. Then Bo Qu et al. [14], made hybrid mixtures while using 30% CK, 32.5% GGBFS, 32.5% FA, and 5% solid activator, obtaining results at 28 days of curing of 35MPa, however in this investigation the activator used was not specified. Following that, the results that were obtained in this research were very similar to those previously reported by other authors, so this project takes great relevance.

### 3.2. X-ray Diffraction

**Figure 2:**
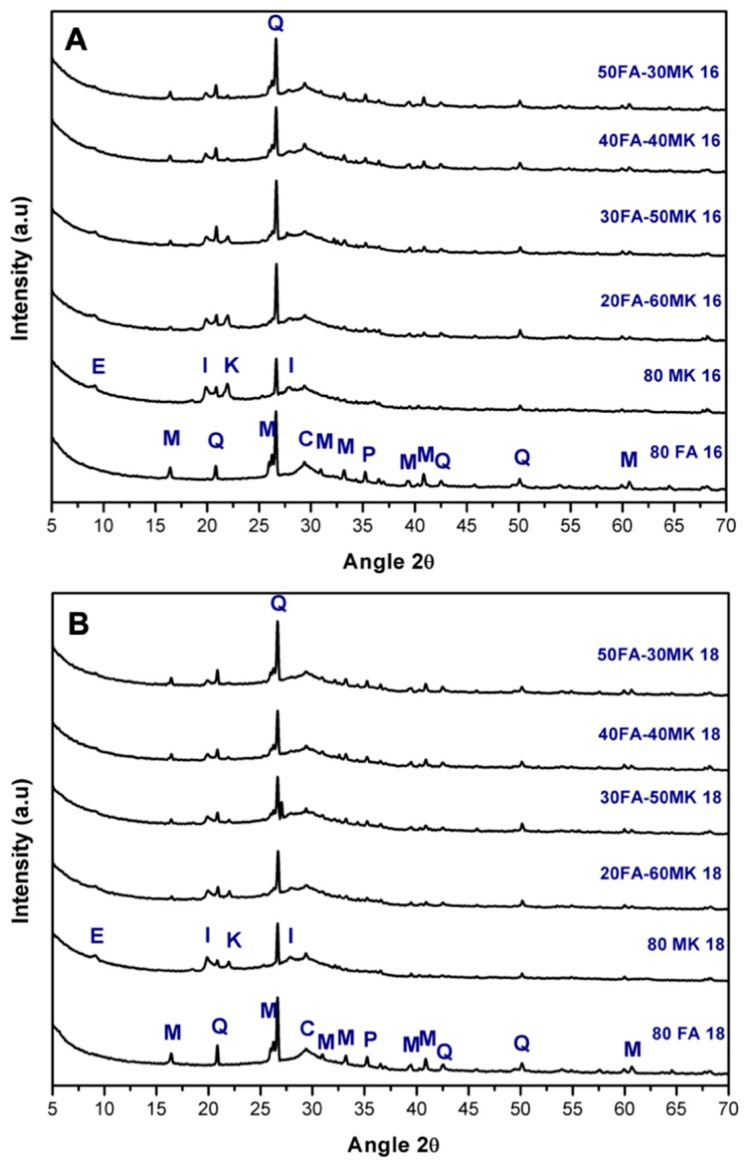
X-ray diffraction patterns of the systems cured for 90 days of: (**A**) activator with 16% Na_2_O and (**B**) activator with 18% Na_2_O. Crystalline phases observed: Q: Quartz, M: Mullite, K: Cristobalite, P: Portlandite, I: Illite, E: Ettringite, C: Calcite.

Figure 2 shows the X-ray diffraction patterns of all systems with 16% and 18% Na_2_O of activation, cured at 90 days. In the figure is possible to observe an amorphous halo between 20 and 40° 2θ, which is associated with the amorphous fraction of the FA and MK. It can also be observed that the main crystalline phases of CK, such as alite and belite, are not observed in the diffraction patterns; with this, it can be confirmed that these phases reacted during the hydration process of all the systems. In the diffraction patterns from Figure 2, it is possible to observe that when the presence of FA increases, the reflection of the crystalline phases from the FA are still present in the systems, as mullite and quartz. This could be attributed to the fact that with the alkaline activation the crystalline phases remain unchanged, and only the amorphous phase of the original fly ash is affected [30].

The presence of portlandite was observed in systems with FA, with a low-intensity reflection; this was not observed when using MK. This might be due to the difference in pozzolanic behaviors of both, where MK has been reported to be more reactive than FA [28].

A very small reflection is also observed in the angular position of 7° 2θ, for samples with a high MK content; this is due to the formation as hydration product of the secondary phase ettringite, it should be noted that the presence of this phase has been reported for this type of cement [31,32], the responsibility for the mechanical strength of the systems studied is an amorphous gel formed as the main hydration product. Wu and Naik T. et al. [33] found that a beneficial effect occurs due to the capacity of this phase to increase its volume up to 160%, since it densifies the matrix increasing the strength of the systems. The illite phase observed in the patterns is a phase that has been reported as an inert phase in MK [1,11], the intensity of reflection of this phase is higher in the systems with more MK content, which suggests the lack of reaction of this phase. The crystalline quartz phase remains unchanged during the alkaline activation process when it comes from both MK, and FA raw materials [34], because this phase does not participate in the geopolymerization reactions [35].

### 3.3. Fourier Transformed Infrared Spectroscopy

**Figure 3:**
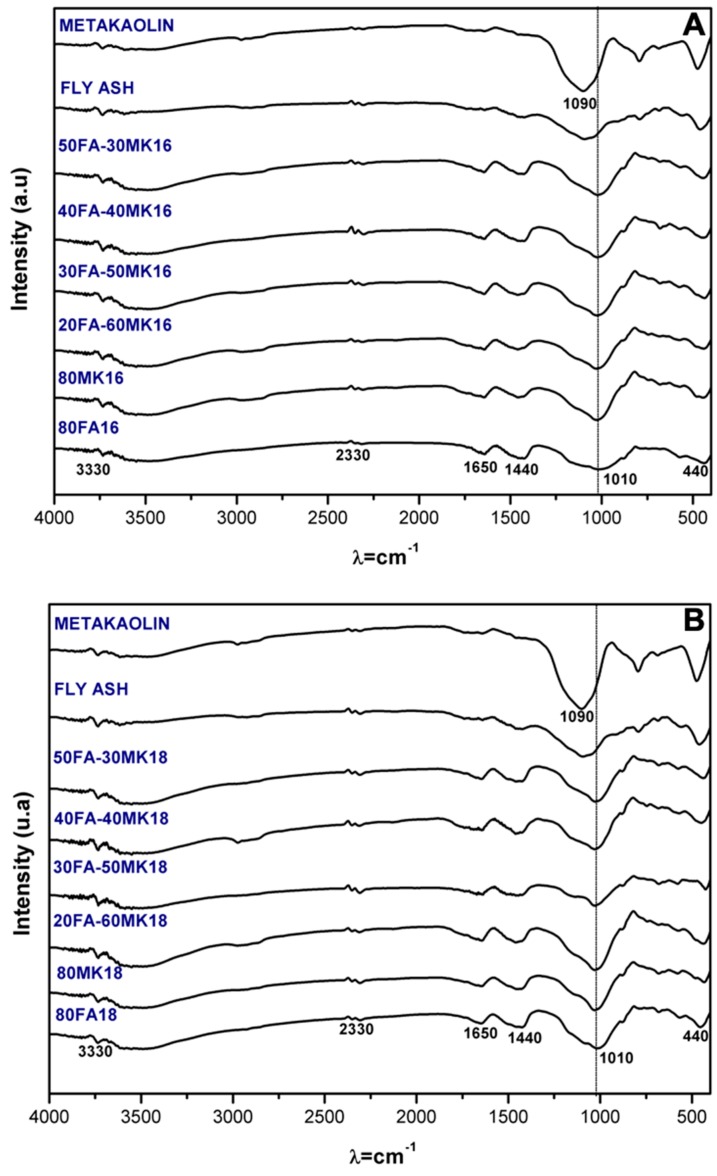
Fourier transform infrared spectroscopy spectra of all the systems cured at 90 days with alkaline activator with a content of (**A**) 16% Na_2_O and (**B**) 18% Na_2_O.

The Fourier transformed infrared spectra of all the systems cured at 90 days are presented in Figure 3. The spectra were interpreted based on literature and past research on hybrid cements. These were compared with the raw material spectra. Several important features are noteworthy, including the inhibition of the water evaporation, associated with the –H–O–H– vibration bands in the region 1640–1660 cm^−1^ corresponding to free water in the system [36], and between 2200–3600 cm^−1^ are the characteristic bands of the –OH– type (H-bonded) [37].

Is worth to notice that in all the systems, can clearly be observed bands at the region around 1490–1440 cm^−1^ related by bonds type O–C–O with vibrations of asymmetrical tension of CO_3_^2−^ can be clearly observed [38], which could be related with the carbonation of the alkali activation solution, reported in other studies [39]. Nevertheless, in some cases could be attributed to the formation of sodium carbonate, which has been observed in similar samples [40,41]. There is an agreement in the literature that the structural evolution of most of the alkali activation is concentrated in the main asymmetric band at around 1200–900 cm^−1^ that originates from bonds T–O–T, where T is Si or Al. In silicate glasses the Si–O–Si band is associated with the Q^n^ units, the band appears at 1200 cm^−1^ (n = 4), 1100 cm^−1^ (n = 3), 950 cm^−1^ (n = 2), 900 cm^−1^ (n = 1), and 850 cm^−1^ (n = 0), however, when the replacement of Si by Al increases, the band moves towards lower values [42]. Following that, in the activation of the raw material (MK) it can be observed a shift of the spectrum to lower frequencies in the bands close to 1093 cm^−1^, this correlates to lower frequencies up to 1012 cm^−1^ in activated metakaolin. This shift has been reported as an indicator of the silicates and aluminosilicates dissolution [38,43], promoting reactions.

The figure also shows that a band that is located at 1090 cm^−1^ in the raw materials as MK and FA, which corresponds to the asymmetric vibrations of the T–O bonds (T = Al, Si) [44], suffers a slippage to 1010 cm^−1^ after being activated with the equivalent Na_2_O, this phenomenon has been previously reported in other investigations and it is attributed to the alkaline activation and dissolution of the aluminosilicates present in the raw materials and the formation of gels [45]. In addition, it is possible to observe the appearance of bands located in the 980–1000 cm^−1^ region; this could be attributed to unreacted clinker [46], due to an increase in the wavelength of the asymmetric stretching tension bands of Si–O bonds in C–S–H gel, which is correlated to a high degree of polymerization that promotes the formation of reaction products (that could also be associated with the formation of C–A–S–H) [36].

The vibrations modes that were observed at around 400 and 600 cm^−1^ could be associated with the Si–O–Si and Al–O–Si bonds. A band around 450 cm^−1^ related with Si–O–Si and O–Si–O bonds was also observed; its intensity has been related to the crystallinity of the material [47]; in this case, the intensity was reduced in all of the systems when compared to the unreacted MK.

In the case of hybrid cements, there is evidence of the formation of new hydration products with a lower degree of polymerization (N–A–S–H/C–S–H/S–H/C–A–S–H) [12]. The characteristic bands corresponding to the Si–O–Si and O–Si–O links are observed between 420–470 cm^−1^.

### 3.4. Scanning Electron Microscopy

**Figure 4:**
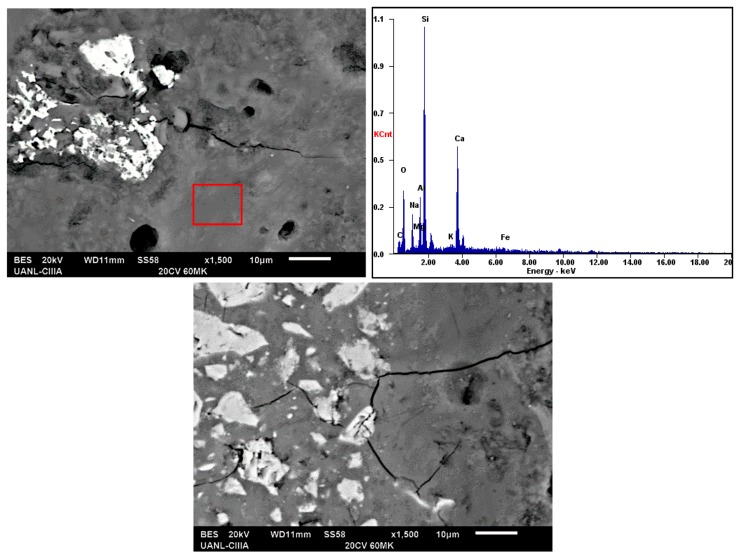
Micrographs of systems cured at 90 days with 16% Na_2_O as alkaline activator.

**Figure 5:**
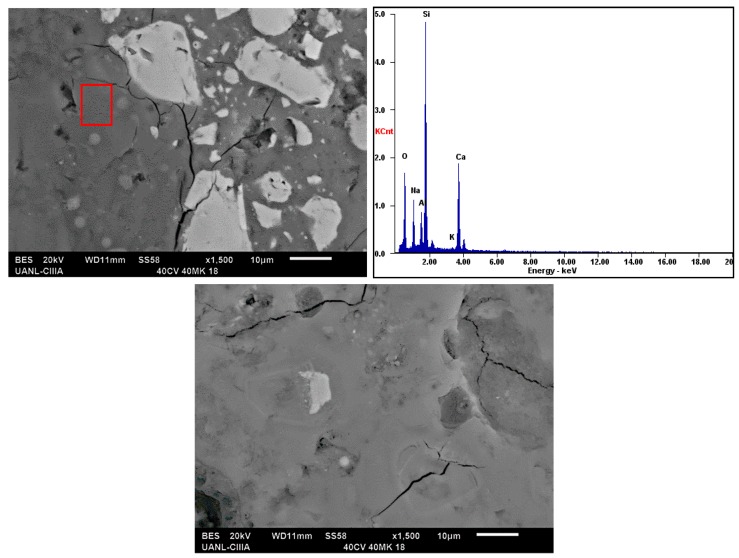
Micrographs of systems cured at 90 days with 18% Na_2_O as alkaline activator.

Figure 4 and Figure 5 show the micrographs of the system with a high compressive strength (20FA–60MK activated with 16% Na_2_O and 40FA-40MK activated with 18% Na_2_O); samples were cured at room temperature for 90 days and characterized in the SEM using backscattered electron images to analyze the compositional contrast and by Energy-dispersive X-ray spectroscopy (EDS) to study the chemical composition of the hydration products. Both of the figures show microstructures with unreacted spherical particles, which are attributed to FA and irregular particles that are attributed to MK [48]. A homogeneous matrix with low porosity, which was reflected in a high mechanical strength, was also observed. The main features that are observed are those commonly presented in cement pastes and geopolymers: (a) with a fraction of unreacted raw material, as can be seen in the semi-white regions in the samples; (b) reaction products that are gray due to the water content in them; and, (c) porosity (darker areas) dispersed throughout the microstructure. When comparing both figures, is possible to notice more porosity in Figure 4, which could be related to lower compressive strength. It is also possible to observe a more compact hydration products matrix in the presence of more activator (Figure 5), which could be an indicative of the improvement of the properties with more activator, at least with the quantities that are used in this work and in this sample. The reduction in the porosity could be due to the filler effect MK and its higher reactivity and the formation of more hydration products in the presence of the activator (Figure 5).

The EDS results showed in both of the figures indicated that the matrix was mostly composed of elements, such as Si, Ca, Al, Na, which is indicative that the matrix is constituted of two types of gels already reported: (a) one rich in calcium, silicon, and aluminum identified as C–A–S–H. This gel could be similar to that identified in the hydration of an OPC, but with Al in its composition, and (b) a gel composed of sodium, silicon, and aluminum, known as N–A–S–H [1,49].

The existence of these two gels and their equilibrium is possible [23,50] due to the ion exchange capacity in the alkaline aluminosilicate gel, where the Ca^2+^ ion coming from the CK displaces the Na^+^ ions of the structure, demonstrates that the Ca^2+^ ion has the capacity to replace between 67% and 100% of the Na^+^ ion. However, the amount of Ca^2+^ ions are not enough to degrade the Na^+^ ions and go from N–A–S–H gel to C–A–S–H gel; for this reason, a hybrid gel rich in silicon and aluminum is formed, and it incorporates calcium and sodium in its structure, which can be called C–(N)–A–S–H cementitious gel. This has been reported by several authors in hybrid cements [11].

According to the EDS results, the ratios that were obtained from the hydration products from the matrices, as in Figure 4, indicated the following: (a) CaO/SiO_2_ ratio of 0.28, which was between 0 and 0.3, and (b) Al_2_O_3_/SiO_2_ of 0.13, which was between the values of 0.05 and 0.43; these ranges of values have been reported in the literature and confirm the presence of the amorphous gel C–(N)–A–S–H [26,51]. This gel has a tridimensional structure that could improve the mechanical strength in this type of materials, but it is still under study. On the other hand, in Figure 5 the results were very similar; with ratios of (a) CaO/SiO_2_ ratio of 0.22 and (b) Al_2_O_3_/SiO_2_ of 0.23; those values were in line with the information previously discussed.

Additionally, when comparing the CaO/SiO_2_ and Al_2_O_3_/SiO_2_ ratios of the raw materials as metakaolin with values of CaO/SiO_2_ = 0.003, and Al_2_O_3_/SiO_2_ = 0.585; and, fly ash with values of CaO/SiO_2_ = 0.046 and Al_2_O_3_/SiO_2_ = 0.4109; an increase in the CaO/SiO_2_ ratio and a decrease in the Al_2_O_3_/SiO_2_ ratio can be noticed in the resulting hydration products matrices, thus indicating the presence of the gels previously described.

## 4. Discussion

Previous research reports that use SCMs, such as FA and MK, not only contributes to the reduction of environmental pollution, but also makes a positive contribution to the sustainability and durability of concrete and mortars [52,53,54]. Following from that, the purpose of this investigation was to examine the effects of partially replacing OPC with massive-generated materials from different production sources that would create a cementitious composite material with similar properties to the currently marketed binder, but without all of the detrimental effect to the environment. One of the most important points was to determine the optimal proportion of materials to meet the proposed goals. However, another fundamental aspect to discuss was the need to use high percentages of alkaline activators that would be capable of making the involved materials react, which is not easy when considering the complexity of the species, as well as the variety of chemical compounds that exist commercially and are likely to meet this objective.

The efficacy of sodium hydroxide and sodium silicate was tested on the treated species after extensive experimentation and analysis of the effect of the selected activators. The best proportion of these compounds was found to be 18% equivalent to Na_2_O, however the very complex characteristics of the systems showed variations with the Na_2_O equivalent content.

There is a selective effect of the activators, which could be attributed to the result of the differences in the chemical composition of the replacement materials, according to Shy and Day et al. [31], who analyzed the effects of activators and replacement materials in the hydration and compressive strength products of alkaline activated composite cements. However, the selectivity does not lie primarily in the chemical composition of the replacement materials, but basically in the reactivity of the constituents determined by their glass phase content, according to the analysis. Another reason why the effectiveness of the activators was different depends on their ability to release the Na^+^ ion, which has the capacity to act as a network modifier [55], and it is determined on the ability of the activator to dissolve in the mixing water, and the subsequent release of the ion of interest.

On the other hand, as previously discussed, MK promotes the formation of ettringite, which produces expansion and, in some cases, cracking and fracture of the specimens. However, it is possible that the expansion generated caused the densification of the cementitious matrix reducing the presence of very large pores, leading to the elimination of porosity or the presence of smaller pores and, therefore, the improvement of the mechanical properties. Nevertheless, it is important to discuss the effect of each of the replacement materials on the cementitious matrix of the systems analyzed in this paper.

**Figure 6:**
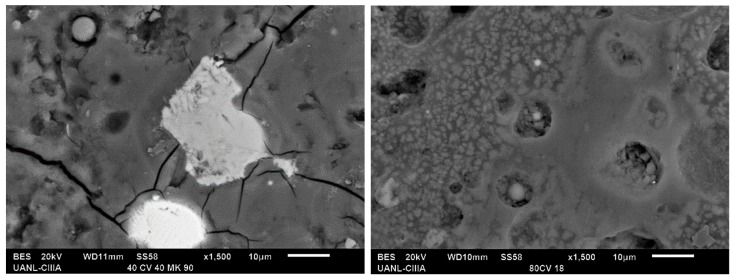
Fly ash particles in the pores of the cementitious matrix.

*Fly ash*: One of the main advantages of using FA is the ability to improve the flowability of the pastes due to its plasticizing characteristics that result from the spherical morphology. According to SEM observations, there was no contribution to the formation of C–S–H gel, since there was no interaction between this material and the alkaline activators used. Even though the ash did not react as expected, its presence was not trivial, since, it promoted the reduction of the porosity of the cementitious matrices due to its variety of spherical sizes and shapes (micro-filling effect). In Figure 6, it can be seen how the FA particles even increase the percentage of the presence of activators that are still present and in the pores of the matrix.

**Figure 7:**
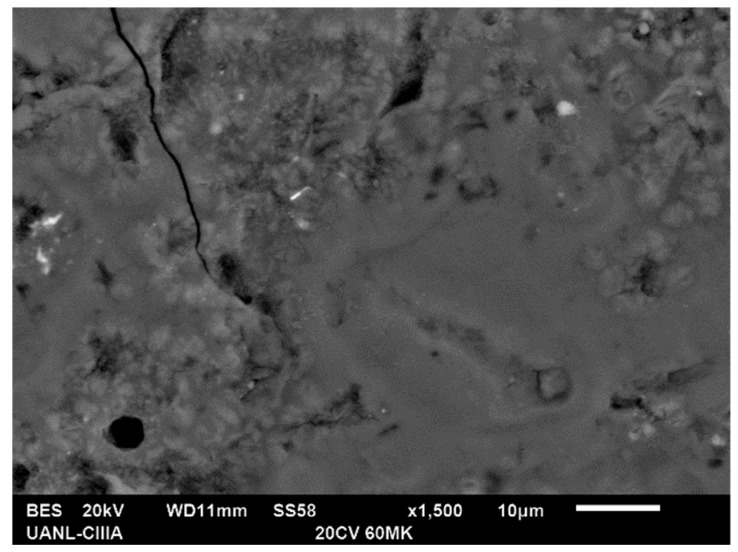
Cementitious matrix with high metakaolin content.

*Metakaolin*: Due to its small particle size there was a significant improvement in the reduction of porosity, see Figure 6 and Figure 7. The existence of an effective pozzolanic reaction was corroborated according to the consumption of portlandite found in XRD and the effect of acting as nucleation sites.

From the results that are presented in this paper, it is possible to indicate that FA and MK formed an excellent duo, since very favorable results were obtained in some systems.

## 5. Conclusions

The results indicated that the synthesis of hybrid cements while using MK and FA as supplementary cementitious materials and a percentage of less than 30% of OPC clinker was successful, while utilizing the activation of the pastes with the combination of alkaline activators, such as sodium hydroxide and sodium silicate, and obtaining mechanical properties that were similar than those of an OPC.

According to the results of the compression test, the system with the best mechanical properties at early ages was the system with 20FA-60MK 16; however, for the systems with higher FA substitution, they were higher when a percentage of 18% of Na_2_O was used, that is to say, the FA is favored with the presence of greater amount of alkaline activator. In systems with a greater presence of MK, the highest values of resistance to compression were found when using 16% Na_2_O, the reason why it can be said that for the activation of the metakaolin it is not required higher quantities of alkaline activator.

The percentage used in this research of OPC clinker (20%) guarantees the reaction of almost all the main crystalline phases of the clinker, as they were not observed in the diffraction of the analyzed pastes. The portlandite phase that is present in the systems with the highest FA content is attributed to the difference in pozzolanic activity between MK and ash, with MK being a material with higher pozzolanic activity.

Cementitious gels with a lower degree of polymerization (N–A–S–H/C–(N)–A–S–H) were identified based on the relationships of the oxides present in the matrices of all the systems that were synthesized in this research, and while comparing them with those already reported in the literature for this type of amorphous gel.

## Figures and Tables

**Table 1:** Chemical composition and physical properties of fly ash (FA), metakaolin (MK), and clinker (CK), obtained while using X-ray fluorescence.

Oxide (wt.%)	FA	MK	CK
SiO_2_	59.57	59.59	22.95
Al_2_O_3_	24.48	34.91	5.7
Fe_2_O_3_	3.94	1.52	2.75
CaO	2.77	0.20	68.55
MgO	0.78	0.05	-
TiO_2_	0.84	0.98	-
Na_2_O	0.60	0.07	-
K_2_O	1.02	0.8	-
SO_3_	0.42	0.41	-
Density (g/cm^3^)	2.19	2.24	3.25
Blaine (cm^2^/g)	4462	5350	5234

**Table 2:** Chemical composition of sodium silicate (Na_2_SiO_3_) and sodium hydroxide (NaOH) that were obtained by chemical analysis and provided by the manufacturer, respectively.

Oxide (wt.%)	Na_2_SiO_3_	NaOH
SiO_2_	37.86	-
Na_2_O	11.72	-
H_2_O	50.42	-
NaOH	-	98.7

**Table 3:** Percentage of precursor powders in pastes.

System	FA	MK	CK	Na_2_O	w/s *
80FA 16	80	-	20	16	0.65
80MK 16	-	80	20	16	0.65
20FA-60MK 16	20	60	20	16	0.65
30FA-50MK 16	30	20	20	16	0.65
40FA-40MK 16	40	40	20	16	0.65
50FA-30MK 16	50	30	20	16	0.65
80FA 18	80	-	20	18	0.65
80MK 18	-	80	20	18	0.65
20FA-60MK 18	20	80	20	18	0.65
30FA-50MK 18	30	50	20	18	0.65
40FA-40MK 18	40	40	20	18	0.65
50FA-30MK 18	50	30	20	18	0.65

* w/s: water to solid ratio.

## References

[1] García-Lodeiro I., Fernández-Jiménez A., Palomo A. (2013). Cementos híbridos de bajo impacto ambiental: Reducción del factor clinker. Rev. Alconpat.

[2] Torres-Carrasco M., Puertas F. (2017). Alkaline activation of different aluminosilicates as an alternative to OPC: Alkali activated cements or geopolymers. Rev. Ing. Constr.

[3] Ke J., Mcneil M., Price L., Khanna N.Z., Zhou N. (2013). Estimation of CO_2_ emissions from China’ s cement production: Methodologies and uncertainties. Energy Policy.

[4] Ludwig H.M., Zhang W. (2015). Research review of cement clinker chemistry. Cem. Concr. Res..

[5] Lothenbach B., Scrivener K., Hooton R.D. (2011). Supplementary cementitious materials. Cem. Concr. Res..

[6] Fernández-Jiménez A., Puertas F., Sobrados I., Sanz J. (2003). Structure of calcium silicate hydrates formed in alkaline-activated slag: Influence of the type of alkaline activator. J. Am. Ceram. Soc..

[7] Duxson P., Fernández-Jiménez A., Provis J.L., Lukey G.C., Palomo A., van Deventer J.S.J. (2007). Geopolymer technology: The current state of the art. J. Mater. Sci..

[8] Fernández-Jiménez A., Palomo A., Sobrados I., Sanz J. (2006). The role played by the reactive alumina content in the alkaline activation of fly ashes. Microporous Mesoporous Mater..

[9] Palomo A., Alonso S., Fernandez-Jiménez A. (2004). Alkaline activation of fly ashes: NMR study of the reaction products. J. Am. Ceram. Soc..

[10] Garcia-Lodeiro I., Palomo A., Fernández-Jiménez A. (2015). An overview of the chemistry of alkali-activated cement-based binders. Handbook of Alkali-Activated Cements, Mortars and Concretes.

[11] Fernández-Jiménez A., Zibouche F., Boudissa N., García-Lodeiro I., Abadlia M.T., Palomo A. (2013). “Metakaolin-slag-clinker blends.” the role of Na+or K+as alkaline activators of theses ternary blends. J. Am. Ceram. Soc..

[12] Rivera J.F., de Gutierrez R.M., Mejia J.M., Gordillo M. (2014). Hybrid cement based on the alkali activation of by-products of coal. Rev. La Constr..

[13] Lodeiro I.G., Macphee D.E., Palomo A., Fernández-Jiménez A. (2019). Effect of alkalis on fresh C–S–H gels. FTIR analysis. Cem. Concr. Res..

[14] Qu B., Martin A., Pastor J.Y., Palomo A., Fernández-Jiménez A. (2016). Characterisation of pre-industrial hybrid cement and effect of pre-curing temperature. Cem. Concr. Compos..

[15] Habert G., de Lacaillerie J.B.D., Roussel N. (2011). An environmental evaluation of geopolymer based concrete production: Reviewing current research trends. J. Clean. Prod..

[16] Snellings R., Mertens G., Elsen J. (2012). Supplementary cementitious materials. Rev. Mineral. Geochem..

[17] Rattanasak U., Chindaprasirt P. (2019). Influence of NaOH solution on the synthesis of fly ash geopolymer. Miner. Eng..

[18] Emeritus T. (1997). Cement Chemistry.

[19] Badogiannis E., Tsivilis S., Papadakis V., Chaniotakis E. (2002). The effect of metakaolin on concrete properties. International Congress on Challenges of Concrete Construction.

[20] Rashad A.M. (2013). Metakaolin as cementitious material: History, scours, production and composition-A comprehensive overview. Constr. Build. Mater..

[21] Moodi F., Ramezanianpour A.A., Safavizadeh A.S. (2011). Evaluation of the optimal process of thermal activation of kaolins. Sci. Iran..

[22] Alonge O.R., Ramli M.B., Lawalson T.J. (2017). Properties of hybrid cementitious composite with metakaolin, nanosilica and epoxy. Constr. Build. Mater..

[23] Yip C.K., Lukey G.C., van Deventer J.S.J. (2005). The coexistence of geopolymeric gel and calcium silicate hydrate at the early stage of alkaline activation. Cem. Concr. Res..

[24] Palomo A., Fernández-Jiménez A., Kovalchuk G., Ordoñez L.M., Naranjo M.C. (2007). Opc-fly ash cementitious systems: Study of gel binders produced during alkaline hydration. J. Mater. Sci..

[25] García-lodeiro I., Maltseva O., Palomo Á., Fernández-jiménez A.N.A. (2012). Hybrid alkaline cements. Part I: Fundamentals. Rev. Romana De Mater..

[26] Garcia-Lodeiro I., Palomo A., Fernández-Jiménez A., MacPhee D.E. (2011). Compatibility studies between N-A-S-H and C-A-S-H gels. Study in the ternary diagram Na_2_O-CaO-Al_2_O_3_-SiO_2_-H_2_O. Cem. Concr. Res..

[27] Barnett S.J., Soutsos M.N., Millard S.G., Bungey J.H. (2006). Strength development of mortars containing ground granulated blast-furnace slag: Effect of curing temperature and determination of apparent activation energies. Cem. Concr. Res..

[28] Bijen J. (1996). Benefits of slag and fly ash. Constr. Build. Mater..

[29] Gorokhovsky A.V., Mendoza G., Fuentes A.F. (2003). Effect of geothermal waste on strength and microstructure of alkali-activated slag cement mortars. Cem. Concr. Res..

[30] Palomo A., Criado M. (2006). Alkali activated fly ash binders. A comparative study between sodium and potassium activators. Mater. Construcción.

[31] Shi C., Day R.L. (2003). Pozzolanic reaction in the presence of chemical activators: Part II–Reaction products and mechanism. Cem. Concr. Res..

[32] Lee C.Y., Lee H.K., Lee K.M. (2003). Strength and microstructural characteristics of chemically activated fly ash–cement systems. Cem. Concr. Res..

[33] Wu Z., Naik T. (2003). Chemically Activated Blended Cements. ACI Mater. J..

[34] Fernández-Jiménez A., Monzó M., Vicent M., Barba A., Palomo A. (2008). Alkaline activation of metakaolin-fly ash mixtures: Obtain of zeoceramics and zeocements. Microporous Mesoporous Mater..

[35] Chen L., Wang Z., Wang Y., Feng J. (2016). Preparation and properties of alkali activated metakaolin-based geopolymer. Materials.

[36] Yu P., Kirkpatrick R.J., Poe B., McMillan P.F., Cong X. (1999). Structure of calcium silicate hydrate (C-S-H): Near-, Mid-, and Far-infrared spectroscopy. J. Am. Ceram. Soc..

[37] Lee W.K.W., van Deventer J.S.J. (2003). Use of infrared spectroscopy to study geopolymerization of heterogeneous amorphous aluminosilicates. Langmuir.

[38] Rees C.A., Provis J.L., Lukey G.C., van Deventer J.S. (2007). Attenuated total reflectance fourier transform infrared analysis of fly ash geopolymer gel aging. Langmuir.

[39] Gomez-zamorano L.Y., Vega-cordero E., Struble L. (2016). Composite geopolymers of metakaolin and geothermal nanosilica waste. Constr. Build. Mater..

[40] Abdollahnejad Z., Hlavacek P., Miraldo S., Pacheco-Torgal F., de Aguiar J.L.B. (2014). Compressive strength, microstructure and hydration products of hybrid alkaline cements. Mater. Res..

[41] Hamidi R.M., Man Z., Azizli K.A. (2016). Concentration of NaOH and the effect on the properties of fly ash based geopolymer. Procedia Eng..

[42] Duxson P., Lukey G.C., van Deventer J.S.J. (2006). Thermal evolution of metakaolin geopolymers: Part 1–Physical evolution. J. Non-Cryst. Solids.

[43] Bass J.L., Turner G.L. (1997). Anion distributions in sodium silicate solutions. Characterization by ^29^ SI NMR and infrared spectroscopies, and vapor phase osmometry. J. Phys. Chem. B..

[44] Fernández-Jiménez A., Palomo A. (2005). Mid-infrared spectroscopic studies of alkali-activated fly ash structure. Microporous Mesoporous Mater..

[45] Criado M., Fernández-Jiménez A., Palomo A. (2007). Alkali activation of fly ash: Effect of the SiO_2_/Na_2_O ratio. Microporous Mesoporous Mater..

[46] Bernal S.A., Provis J.L., Rose V., Mejía R., Gutierrez D. (2011). Evolution of binder structure in sodium silicate-activated slag-metakaolin blends. Cem. Concr. Compos..

[47] de Silva P., Sagoe-crenstil K. (2008). Medium-term phase stability of Na_2_O–Al_2_O_3_–SiO_2_–H_2_O geopolymer systems. Cem. Concr. Res..

[48] Bernal S.A., Provis J.L., Fernández-Jiménez A., Krivenko P.V., Kavalerova E., Palacios M., Shi C. (2014). Binder chemistry–high-calcium alkali-activated materials. Alkali activated materials.

[49] García-Lodeiro I., Fernández-Jiménez A., Palomo A. (2013). Variation in hybrid cements over time. Alkaline activation of fly ash-OPC blends. Cem. Concr. Res..

[50] Alonso S., Palomo A. (2001). Calorimetric study of alkaline activation of calcium hydroxide–metakaolin solid mixture. Cem. Concr. Res..

[51] García-Lodeiro I., Fernández-Jiménez A., Palomo A., MacPhee D.E. (2010). Effect of calcium additions on N-A-S-H cementitious gels. J. Am. Ceram. Soc..

[52] Khan H.A., Khan M.S.H., Castel A., Sunarho J. (2018). Deterioration of alkali-activated mortars exposed to natural aggressive sewer environment. Constr. Build. Mater..

[53] Fernández-jiménez A., Palomo A. (2009). Propiedades y aplicaciones de los cementos alcalinos. Rev. Ing. De Construcción.

[54] Bernal S.A., de Gutiérrez R.M., Ruiz F., Quiñones H., Provis J.L. (2012). Desempeño a temperaturas altas de morteros y hormigones basados en mezclas de escoria/metacaolín activadas alcalinamente. Mater. Construcción.

[55] Atomic T.H.E., Glass I.N. (1932). Nippon Seramikkusu Kyokai gakujutsu ronbunshi. J. Am. Chem. Soc.

